# Clinical efficacy of submucosal injection of triamcinolone acetonide in the treatment of type II/III interstitial cystitis/bladder pain syndrome

**DOI:** 10.1186/s12894-020-00597-3

**Published:** 2020-03-30

**Authors:** Tao Jiang, Xiaozhou Zhou, Zhipeng Chen, Tailin Xiong, Jian Fu, Zhengchao Liu, Dishi Yan, Zhansong Zhou, Wenhao Shen

**Affiliations:** Department of Urology, Urology Institute of PLA, Southwest Hospital, Third Military Medical University (Army Medical University), Gao Tanyan Street 29#, Sha Pingba, Chongqing, 400038 China

**Keywords:** Bladder pain syndrome, Hunner’s lesion, Interstitial cystitis, Triamcinolone

## Abstract

**Background:**

To evaluate the efficacy of submucosal injection of triamcinolone acetonide for the treatment of type II/III interstitial cystitis/bladder pain syndrome.

**Methods:**

A retrospective analysis of the clinical data of type II/III interstitial cystitis/bladder pain syndrome patients treated in our department from April 2016 to August 2018 was conducted, and changes in International Prostate Symptom Scores and the Pelvic Pain and Urgency/Frequency symptom scale after surgery were evaluated to explore factors that may affect patient outcomes.

**Results:**

A total of 27 female patients and 8 male patients were enrolled, with type II patients accounting for 62.9% of the sample, and the median follow-up duration was 31 months (range: 12–40 months). Twenty-two patients (74.3%) had significantly improved questionnaire scores at 4 weeks postoperatively. Treatment efficacy was sustained for at least 1 year in 15 patients, and persistent effectiveness was noted in 10 (28.6%) patients. Patients with an advanced age (*p* = 0.015), high pain scores (*p* = 0.040), and higher International Prostate Symptom Scores (*p* = 0.037) and Pelvic Pain and Urgency/Frequency symptom scale scores (*p* = 0.020) were more likely to benefit from submucosal injection of triamcinolone acetonide. Gender, disease duration, and the presence of Hunner’s lesions had no predictive value for treatment outcomes.

**Conclusion:**

Submucosal injection of triamcinolone acetonide can improve the clinical symptoms and quality of life in both men and women with type II/III interstitial cystitis/bladder pain syndrome. Patients with an advanced age and more severe interstitial cystitis/bladder pain syndrome related symptoms may benefit more from triamcinolone acetonide injection.

## Background

Interstitial cystitis/bladder pain syndrome (IC/BPS) is a clinical syndrome with an unclear cause. It mainly manifests as pain in the suprapubic area or discomfort with bladder filling, which can be alleviated with urination. IC/BPS is often accompanied by frequent urination, urgency, and nocturia and is more common in middle-aged women. IC/BPS seriously affects the quality of life of patients [1]. The etiology and pathophysiology of this disease are still not fully understood, and readily available and effective treatment methods are currently lacking in clinical practice.

The European Society for the Study of Interstitial Cystitis (ESSIC) divided IC/BPS into the following three major types based on changes in the bladder mucosa after cystoscopy and/or bladder hydrodistention in IC/BPS patients: type I: normal bladder mucosa; type II: glomerulations in the bladder mucosa; and type III: visible Hunner’s lesions in the bladder mucosa with or without glomerulations. Type II and type III account for 30 ~ 60% of all IC/BPS cases [2].The American Urological Association (AUA) IC/BPS guideline of recommends endoscopic laser ablation/ electrocauterization of Hunner’s lesions or injection of triamcinolone acetonide (TA) into lesions as alternative treatments for Hunner’s subtype of IC/BPS [3].

Cox and other researchers have reported treating female patients with type III IC/BPS with submucosal TA injection [4]. Whether this treatment is effective for male patients remains unclear, and no studies have reported treating type II IC/BPS using the same method [4, 5]. This study is the first to include male patients and type II IC/BPS patients. With at least 1 year of follow-up for each patient, this study showed the efficacy of TA for the treatment of IC/BPS patients with intravesical Hunner’s lesions and/or submucosal hemorrhage in our center, and relevant factors that may affect the treatment efficacy were analyzed.

## Methods

The medical records of type II/III IC/BPS patients treated with submucosal TA injection in our hospital from April 2016 to August 2018 were retrospectively analyzed. IC/BPS was diagnosed using the “exclusive diagnosis” method and according to the AUA IC/BPS Guideline [3] and the IC/BPS diagnostic criteria of the ESSIC [2].All patients had a poor response to lifestyle modifications and conservative drug therapy with amitriptyline. Patients who voluntarily underwent cystoscopy and submucosal injection of TA signed an informed consent form. The Institutional Review Board of Southwest Hospital, The Third Military Medical University approved this investigation.

Endoscopic diagnosis and treatment were performed by an experienced specialist. After successful spinal anesthesia, bladder hydrodistention was performed with the patient in the lithotomy position at the pressure of 60 cm H_2_O for 5 min under cystoscopy. After venting, the bladder was filled again, and lesions in the bladder wall were observed and recorded in detail. For patients who did not show obvious abnormalities under cystoscopy, surgery was concluded. For type II/III IC/BPS patients, TA solution was injected into the submucosa at the bladder hemorrhage site and/or into the center of Hunner’s lesions (0.5 ml, 40 mg/ml per point, up to 20 points). After ensuring the absence of active bleeding in the bladder, a 16 Fr double-lumen catheter was inserted. The catheter was removed the next day after surgery.

The patients were assessed with the International Prostate Symptom Score (IPSS) and the Pelvic Pain and Urgency/Frequency (PUF) symptom scale before surgery, and 4 weeks, 6, 12, and 18 months after surgery. If a patient’s symptoms recurred, then he or she returned to the hospital for a timely follow-up.

The results are presented as the means ± standard deviation (SD). Comparisons were performed using the Wilcoxon rank-sum test to analyze scores before and after treatment The Mann-Whitney test was used to analyze factors influencing the treatment effect. Data with *p* values of 0.05 or less was considered statistically significant. A Kaplan-Meier curve was plotted to depict the sustained efficacy duration after the first treatment; Patients for whom no repeat procedure occurred and exhibited persistent effectiveness at the last follow-up were censored. All of the statistical analyses were performed using the Statistical Package for Social Sciences, version 19, for Windows (SPSS, Chicago, IL).

## Results

A total of 35 patients were included in the study, with 8 males (22.9%) and 27 females (77.1%). The patients’ ages ranged from 33 to 80 years, with an average age of 51.7 ± 12.0 years. The average disease duration was 5.2 ± 4.6 years. Twenty-two patients (62.9%) had type II IC/BPS, and 13 patients (37.1%) had type III IC/BPS. All patients were followed up for at least 1 year after the first submucosal injection, with a median follow-up duration of 31 months (range: 12–40 months).

IPSS and PUF scores at 4 weeks after surgery are shown in Table 1. At 4 weeks postoperatively, 26 (74.3%) patients had significant improvements in IC/BPS-related symptoms such as urinary frequency, urgency, and pain. Among these patients, quality of life (QOL) scores decreased by 3.6 points (*p* < 0.0001), IPSS decreased by 17.2 points (p < 0.0001), PUF symptom scores decreased by 8.9 points (p < 0.0001), and PUF bother scores decreased by 4.5 points (p < 0.0001).

**Table 1:** Mean pre-procedure and post-procedure scores for individual questionnaire items and Wilcoxon rank-sum analysis of cohort responses (Wilcoxon rank-sum test)

Question		Pre-procedure Score Mean (SD)	Post-procedure Score Mean (SD)	*p*-value
IPSS	Incomplete Emptying	4.5 (0.7)	2.1 (1.3)	< 0.0001
	Frequency	4.5 (0.7)	2.1 (1.5)	
	Intermittenc	2.1 (2.0)	0.9 (1.2)	
	Urgency	4.4 (0.6)	1.8 (1.4)	
	Weak Stream	2.2 (1.9)	1.0 (1.1)	
	Nocturia	3.7 (0.7)	2.3 (1.0)	
	QOL	5.7 (0.5)	2.1 (2.0)	
	Total Score	31.4 (5.5)	14.2 (9.1)	
PUF	Daytime Voids	3.0 (1.0)	1.0 (1.0)	
	Nighttime Voids	3.7 (0.7)	2.3 (1.0)	
	Pain in Bladder, Pelvis	2.3 (0.9)	0.9 (0.6)	
	Pain Scale	2.1 (0.8)	0.8 (0.7)	
	Urgency after Voiding	2.2 (0.8)	0.9 (0.9)	
	Urgency Scale	2.4 (0.7)	0.8 (1.0)	
	Total Bother Score	7.5 (1.8)	3.0 (3.5)	
	Total Symptom Score	16.6 (3.3)	7.7 (4.9)	
	Total PUF Score	24.1 (4.8)	10.7 (8.1)	

*SD* Standard deviation, *IPSS* International Prostate Symptom Score, *PUF* Pelvic Pain and Urgency/Frequency, *QOL* Quality of life

We further analyzed the clinical characteristics of the patients with a positive therapeutic effect and nonresponsive patients (Table 2). Patients with an advanced age (*p* = 0.015), high pain scores (*p* = 0.040), and high IPSS (*p* = 0.037) and PUF scores (*p* = 0.020) were more likely to benefit from submucosal injection of TA. No difference in the response to treatment was noted for patients with Hunner’s lesions (*p* = 0.644), and male patients had similar treatment response rates to those of female patients (*p* = 0.959). Disease duration had no significant predictive value for treatment efficacy (*p* = 0.516).

**Table 2:** Patient demographics stratified by no response vs symptom relief with analysis of risk factors associated with clinical effect (Mann-Whitney U test)

	No response	Symptom relief	*p*-value
No. patients (%)	9 (25.7)	26 (74.3)	
Age Mean ± SD	41.0 (9.1)	54.5 (11.8)	0.015
No. gender (%)			
Male	2 (22)	6 (23)	0.959
Female	7 (78)	20 (77)	
IC/BPS type (%)			
Type II	6 (66.7)	16 (61.5)	0.644
Type III	3 (33.3)	10 (38.5)	
Symptom history (years) Mean ± SD	4.7 (2.2)	5.4 (5.2)	0.516
Pain Scale Mean ± SD	1.7 (0.5)	2.3 (0.8)	0.040
QOL Mean ± SD	5.4 (0.5)	5.7 (0.5)	0.124
Total IPSS Score Mean ± SD	22.8 (5.0)	26.7 (5.0)	0.037
PUF Bother Score Mean ± SD	6.6 (1.3)	7.9 (1.8)	0.065
PUF Symptom Score Mean ± SD	14.4 (2.4)	17.3 (3.3)	0.019
Total PUF Score Mean ± SD	21.0 (3.2)	25.2 (4.8)	0.020

*SD* Standard deviation, *IC/BPS* Interstitial cystitis/bladder pain syndrome, *IPSS* International Prostate Symptom Score, *PUF* Pelvic Pain and Urgency/Frequency, *QOL* Quality of life

**Fig. 1:**
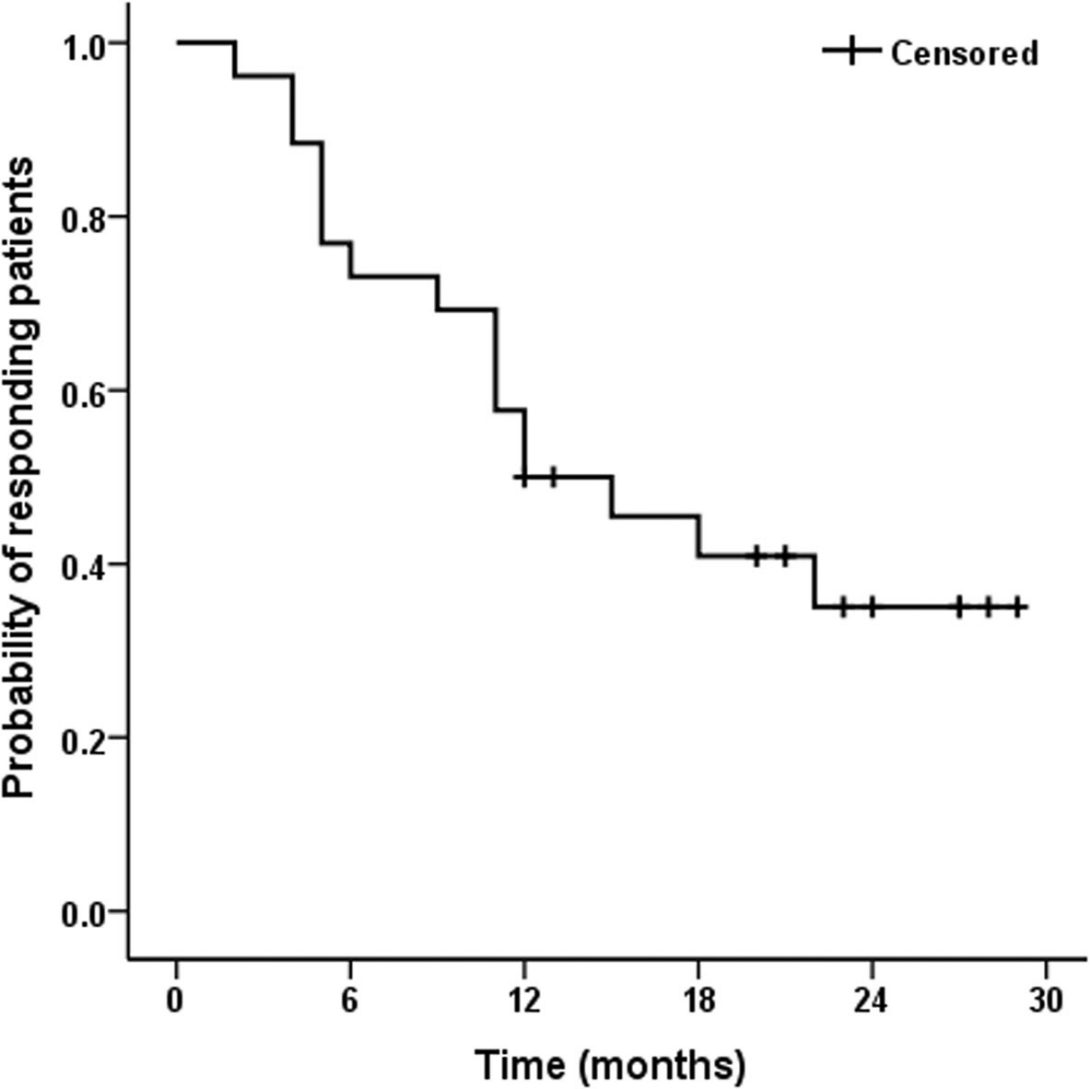
Kaplan-Meier curve shows maintenance time of clinical efficacy after single intramucosal triamcinolone acetonide injection

Fifteen (42.9%) patients were satisfied with symptom control at 1 year after surgery. Their 1-year postoperative scores increased slightly compared with those at 4 weeks after surgery, but no significant difference was found (IPSS: *p* = 0.46, PUF symptom score: *p* = 0.16, and PUF bother score: *p* = 0.60).At the last follow-up, 10 (28.6%) patients had no significant worsening of symptoms. We further analyzed the factors that may have influenced the treatment efficacy for at least 1 year. No clinical parameters that clearly affected patients’ treatment effect duration was found (Online resource Additional file 1: Table S1). The Kaplan-Meier curve shown in Fig. 1 demonstrates the sustained efficacy duration among patients with effective submucosal TA injection.

Seven patients with effective first-time treatment were retreated with submucosal TA injection after symptom recurred. The duration of symptom control after reinjection was basically the same as that with the first injection. No significant intraoperative and postoperative complications were observed in any patients.

## Discussion

IC/BPS is a complex and challenging clinical syndrome. No universally effective treatment is available for this disease. A treatment regimen based on bladder pathology changes is one of the therapeutic directions for IC/BPS. Glomerulations and Hunner’s lesions of the bladder mucosa are typical pathological changes commonly observed with bladder hydrodistention under cystoscopy in patients with IC/BPS, which the ESSIC classifies as type II and III IC/BPS [2]. The IC/BPS guidelines of the AUA recommend surgical removal of ulcers in IC/BPS patients with Hunner’s lesion subtypes [3]. According to studies reported by Payne et al., the average symptom score for IC/BPS patients improved by 76% [6] with electrocautery or removal of Hunner’s lesions under cystoscopy. Some researchers have reported that for refractory IC/BPS patients without ulcers, transurethral electrocautery applied to hemorrhage sites after bladder hydrodistention can also effectively relieve clinical symptoms [7]. Surgical removal of bladder mucosal lesions often requires the lesions to be relatively limited. If the range of Hunter’s lesions is greater than 25% of the bladder mucosal area, then surgical removal is not a suitable treatment option [8]. Regardless of whether electrical resection, electrocautery or laser ablation is performed, potential complications such as bladder perforation and digestive tract injuries may occur, and a larger range of bladder mucosal cauterization may also cause bladder contracture.

Although the etiology of IC/BPS is still unclear, autoimmunity is recognized as one of the main causes of IC/BPS [9]. Supporting evidence demonstrates similar gender and age distributions among IC/BPS patients to those of patients with known autoimmune diseases, and the clinical features of IC/BPS are similar to those of other established autoimmune diseases [10]. Immunosuppressive drugs routinely used to treat autoimmune diseases have yielded positive results in specific IC/BPS patients [11, 12], which also confirms that autoimmunity plays a key role in IC/BPS. Mast cell activation with the release of inflammatory mediators interacting with other inflammatory cells and nervous system also revealed an important role of inflammatory response in the pathogenesis of IC /BPS [2, 13, 14]. TA is a long-acting adrenal corticosteroid with anti-inflammatory, anti-itching and vasoconstricting effects. It has strong and long-lasting anti-inflammatory and anti-allergic effects but a weak water-sodium retention effect. TA is inexpensive and extremely well tolerated when used locally. Cox and other researchers pioneered the submucosal TA injection method for the treatment of refractory Hunner’s lesion-subtype IC/BPS patients and reported satisfactory results^4^. A preliminary study by Rittenberg et al. on IC/BPS patients with Hunner’s lesions and bladder mucosal fissures also showed that TA contributes to symptom control and improves the quality of life of IC/BPS patients [5].

Based on the positive efficacy of electrocautery or electrical resection of bladder mucosal lesions in patients with type II and type III IC/BPS and the significant alleviation of clinical symptoms observed among type III IC/BPS patients with TA injection, we speculate that this treatment is also suitable for type II IC/BPS patients. Although the incidence of IC/BPS is lower in men than in women, IC/BPS in men is not uncommon in clinical practice and is easily misdiagnosed and mistreated. All of the 8 male patients in our study had been diagnosed with chronic prostatitis/chronic pelvic pain syndrome (CP/CPPS) in the past and the therapeutic effect aimed at CP/CPPS with antibiotics, alpha-blockers and other phytotherapeutic agents was poor. For one patient with the symptom of dysuria, concomitant bladder-outlet obstruction was also considered. The maximum flow rate was 11 ml/s with urinary volume 95 ml and the obstruction was ruled out by pressure-flow study with normal result. These male patients complained with pelvic region pain during the urine storage period which could be relieved after urination. The pain is different from CP/CPPS pain, which is usually located in the perineum, penis, testicles or suprapubic region, and often worsens by urination or ejaculation. They were diagnosed with IC/BPS finally. Because the symptoms also could not be relieved by oral taken of amitriptyline. They were enrolled in the study. This study is the first to include male patients with type II IC/BPS, to demonstrate the efficacy of this therapy for Hunner’s lesion-subtype IC/BPS and to explore the efficacy of this treatment in males and type II IC/BPS patients. The IPSS is mainly used to evaluate urinary symptoms, is easy to understand and manage, and can easily and intuitively reflect the negative impact of urinary symptoms on quality of life. By comparing the preoperative and postoperative IPSS and PUF scores, nearly 3/4 of the patients were found to have significant pain relief and reduced urinary tract symptoms at 4 weeks after surgery, and their quality of life improved significantly. No perioperative and long-term complications were noted, and the treatment efficacy was comparable to that reported by researchers such as Cox [4]. Furthermore, we found that submucosal injection of TA at hemorrhage sites can also control the symptoms of IC/BPS, and the efficacy was the same as that for type III IC/BPS. Significant effects can also be achieved in male patients. Among the factors influencing the efficacy outcome, patients with an advanced age and higher IPSS and PUF scores were more likely to experience a good response to treatment.

The current study shows that the duration of the treatment effect of simple bladder hydrodistention for IC/BPS is generally less than 6 months, and the duration of the treatment effect of submucosal injection of TA for IC/BPS is not clear. The longest follow-up time in the current study was only 3 months [5]. We performed a follow-up of all patients for at least 1 year, and 43% of the patients had a one-year remission period in terms of symptom control after this treatment. Patients with symptoms recurrence had a similar remission period after reinjection to that after the first injection. Ten patients still showed effectiveness at the time of the last follow-up, and 5 patients had sustained efficacy for more than 2 years. Whether this treatment can fully control IC/BPS symptoms remains to be further determined.

The main limitations of our study include the retrospective analysis, small sample size, and lack of a control group. Although the time of symptom relief we reported was obviously longer than simple bladder hydrodistention, the efficacy of hydrodistension can’t be ignored. We are aiming at performing a prospective, randomized control study with the groups who will receive saline injections or only bladder hydrodistention at present. The study will reveal more information on the therapeutic effect of submucosal injection of TA for IC/BPS in the future. In addition, The Multidisciplinary Approach to the Study of Chronic Pelvic Pain (MAPP) Network findings [15, 16] indicate that specific clinical factors that are easy to measure – including the pain beyond the pelvis, the presence of nonurological chronic overlapping pain conditions (COPCs) and the severity of bladder-focused symptoms (particularly pain with bladder filling) – have a significant impact on symptom trajectories over time. All the enrolled patients in our study were excluded from COPCs including fibromyalgia, chronic fatigue syndrome and irritable bowel syndrome. However, two patients showed diffuse pain preoperatively and the duration of symptom relief was relatively short after the treatment. More detailed clarification of the location of pelvic pain and of possible concomitant non-urological-associated symptoms according to MAPP consensus should also be considered in the future research.

## Conclusions

Submucosal injection of TA has demonstrated an acceptable safety profile for treating IC/BPS. TA may be effective in controlling symptoms in type II as well as type III IC/BPS, and can improved symptoms and the quality of life of male IC/BPS patients. Patients with an advanced age and more severe IC/BPS-related symptoms may have greater benefits. Further investigation of submucosal injection of TA for the treatment of IC/BPS is warranted with a larger, longer, randomized and controlled trial.

## Supplementary information

**Additional file 1: Table S1.** Patient demographics stratified by the length of time (less than 1 year vs at least 1 year) of symptom relief with analysis of risk factors associated with clinical effect.

## Data Availability

The datasets used and/or analyzed during the current study are available from the corresponding author on reasonable request.

## Abbreviations

AUA: American Urological Association
ESSIC: European Society for the Study of Interstitial Cystitis
IC/BPS: Interstitial cystitis/bladder pain syndrome
IPSS: International Prostate Symptom Scores
PUF: Pelvic Pain and Urgency/Frequency
QOL: Quality of life
TA: Triamcinolone acetonide
