# Supplementary material for: Clinical efficacy of submucosal injection of triamcinolone acetonide in the treatment of type II/III interstitial cystitis/bladder pain syndrome
Source: BMC Urol. 2020 Mar 30;20:36. doi: 10.1186/s12894-020-00597-3 (PMC7106786; doi:10.1186/s12894-020-00597-3)
Supplement: Supplementary file 1 — Additional file 1: Table S1. Patient demographics stratified by the length of time (less than 1 year vs at least 1 year) of symptom relief with analysis of risk factors associated with clinical effect. [file 12894_2020_597_MOESM1_ESM.docx]

**Clinical efficacy of submucosal injection of triamcinolone acetonide in the treatment of type II/III interstitial cystitis/bladder pain syndrome**

Tao Jiang^†^, Xiaozhou Zhou^†^, Zhipeng Chen, Tailin Xiong, Jian Fu, Zhengchao Liu, Dishi Yan, Zhansong Zhou, Wenhao Shen^*^

†Tao Jiang and Xiaozhou Zhou contributed equally to the study.

*Corresponding author.

**Journal：BMC Urology**

**Corresponding author:** Wenhao Shen, Department of Urology, Urology Institute of PLA, Southwest Hospital, Third Military Medical University (Army Medical University), Chongqing, China. E-mail: [chongqingswh@aliyun.com](mailto:chongqingswh@aliyun.com)

Additional Table 1. Patient demographics stratified by the length of time (less than 1-year vs at least 1 year) of symptom relief with analysis of risk factors associated with clinical effect (Mann-Whitney U test)

|  | Symptom relief  less than 1 year | Symptom relief  at least 1 year | p-value |
| --- | --- | --- | --- |
| No. patients (%) | 20 (57.1) | 15 (42.9) |  |
| Age Mean ± SD | 50.4 (12.8) | 53.5 (11.2) | 0.325 |
| No. gender (%) |  |  |  |
| Male | 2 (22) | 6 (23) | 0.731 |
| Female | 7 (78) | 20 (77) |  |
| IC/BPS type (%) |  |  |  |
| Type II | 13(65.0) | 9(60.0) | 0.702 |
| Type III | 7(35.0) | 6(40.0) |  |
| Symptom history (yrs) Mean ± SD | 3.6 (2.4) | 7.3 (5.9) | 0.099 |
| Pain Scale Mean ± SD | 2.0 (0.8) | 2.3 (0.8) | 0.145 |
| QOL Mean ± SD | 5.7 (0.5) | 5.6 (0.5) | 0.633 |
| Total IPSS Score Mean ± SD | 30.1 (5.4) | 32.5 (5.7) | 0.330 |
| PUF Bother Score Mean ± SD | 7.1 (1.5) | 8.2 (2.0) | 0.077 |
| PUF Symptom Score Mean ± SD | 16.0 (3.1) | 17.3 (3.4) | 0.122 |
| Total PUF Score Mean ± SD | 23.1 (4.4) | 25.5 (5.1) | 0.071 |

SD: standard deviation, IC/BPS: Interstitial cystitis/bladder pain syndrome, IPSS: International Prostate Symptom Score, PUF: Pelvic Pain and Urgency/Frequency, QOL: quality of life
